# A qualitative study on cannabis use for harm reduction and pain among veterans enrolled in an SUD treatment program

**DOI:** 10.1186/s12954-026-01412-2

**Published:** 2026-02-19

**Authors:** Traben Pleasant, Sarah Ono, Devan Kansagara, Jennette Lovejoy, Travis Lovejoy, Jessica Wyse

**Affiliations:** 1 Center to Improve Veteran Involvement in Care (CIVIC), US Department of Veterans Affair, 3710 SW US Veterans Hospital Rd. Building 6, Portland, OR 97239 USA; 2 Department of Veterans Affairs, Veterans Rural Health Resource Center, Portland, OR USA; 3 Psychiatry, Oregon Health & Science University, Portland, OR USA; 4 Medicine, Oregon Health & Science University, Portland, OR USA; 5 Communication Studies, University of Portland, Portland, OR USA; 6 Public Health and Preventive Medicine, Oregon Health & Science University, Portland, OR USA

**Keywords:** Cannabis, Harm reduction, Pain, Substance use disorder, Veterans

## Abstract

**Background:**

Two-thirds of adults in the United States (U.S.) find recreational and medical cannabis use acceptable. Evidence shows that cannabis use is an effective treatment for pain, and as a harm reducing therapeutic for Substance Use Disorder (SUD). In contrast to most SUD treatment models, some models allow illegal substance use as a harm reduction strategy, which is the case for the SUD treatment program examined in this study. Little is known about veterans with SUD and their perspectives on cannabis use to treat pain. This study characterizes veteran perspectives on cannabis use to manage chronic pain, and pain-related anxiety and poor sleep, during treatment for non-cannabis SUD.

**Methods:**

Thirty-three U.S. military veterans with a diagnosis of chronic pain, who were receiving care from a Veterans Affairs (VA) SUD treatment program were eligible to participate. Patients with polysubstance use could continue to use substances, as long as they committed to a goal of abstaining from the substance that was the primary focus of their SUD treatment. Most patients had a primary diagnosis of alcohol use disorder (70%), followed by opioid use disorder (18%), and stimulant use disorder (12%). Patients with a primary cannabis SUD were excluded. Patients completed a semi-structured interview. Content analysis identified key narrative themes.

**Results:**

Approximately one-third of patients reported cannabis use during SUD treatment. Motivations for cannabis use were to alleviate pain, pain-related anxiety and poor sleep quality, and viewing cannabis as a harm reducing therapeutic. Approximately half of patients abstained from cannabis due to negative physical and mental effects experienced in the past, fear of returning to use their primary SUD substance, and to avoid the violation of policies or laws prohibiting cannabis use. Some patients who did not use cannabis showed interest in future cannabis use for pain based on recent cannabis science, laymen’s knowledge and curiosity.

**Conclusions:**

Patients with non-cannabis SUD may choose to use cannabis to alleviate chronic pain, pain-related anxiety and poor sleep quality, and as a harm reducing therapeutic during SUD treatment. VA and other federally employed clinicians, particularly in states where cannabis is legal, should prepare to educate patients on the medical evidence, gaps, policies and laws related to cannabis to help patients make more informed healthcare decisions related to cannabis use.

## Background

Attitudes in the United States (U.S.) towards cannabis have significantly shifted in recent decades, as more than two-thirds of adults now accept cannabis use for medicinal and recreational purposes [1]. Thirty-nine states and Washington, D.C. have legalized cannabis for medicinal use, with 24 states permitting recreational use for adults [2–4]. Tetrahydrocannabinol (THC), a psychotropic compound, and its sibling, a non-psychotropic compound called cannabidiol (CBD) are the primary phytocannabinoids in the cannabis plant [5–7]. The cannabis plant contains hundreds of cannabinoids, varying chemical profiles, and unpredictable physical and mental effects on cannabis users, making it difficult to regulate [8]. Within the cannabis industry, this lack of regulation can result in the inaccurate labeling of ingredients and inconsistent cannabis potencies. In a study of cannabis products sold online, only 30% of CBD-advertised products contained the actual reported levels of CBD, while more than 20% of “THC free” products contained THC [9]. Additionally, prior research found that some cannabis labeling leads customers to misunderstand product compositions, and today, sound regulation practices in the cannabis industry across states remains absent [10, 11]. While acceptance of cannabis for medical purposes grows, poor regulation makes prescribing cannabis challenging.

From a medical science perspective, systematic reviews highlight evidence that cannabis may reduce symptoms of anxiety, though its efficacy for mood disorders—including bipolar and depressive disorders—and PTSD remains unclear [12, 13]. Among patients with chronic pain, a 2025 living systematic review conducted by the U.S. Agency for Healthcare Research and Quality of 29 randomized controlled trials and 15 observational studies found that cannabis formulations of equivalent THC and CBD may produce small reductions in pain severity, with low-to-moderate strength of evidence and predominantly in patients with neuropathic pain [14]. This review highlighted that there is insufficient evidence that cannabis improves pain outcomes in other chronic pain syndromes, such as musculoskeletal pain. However, patients who report use of cannabis for chronic pain qualitatively endorse its benefits in improving pain, sleep, and mental and physical functioning,^15^ suggesting that individual patient preferences and experiences, perhaps more than scientific evidence, influences patients’ decisions around cannabis use. It is unclear how such patients might endorse and/or use cannabis to treat pain in the context of substance use disorder treatment.

Substance use disorders (SUD) and chronic pain frequently co-occur [16, 17], and many patients specifically report substance use as a “self-medicating” treatment for chronic pain, including opioids and alcohol [16, 18, 19]. Some patients with a non-cannabis primary SUD may perceive cannabis as an alternative to opioids or other substances to manage pain [20]. Moreover, in the midst of an opioid epidemic, cannabis has been proposed as a harm reduction method to prevent the misuse of opioids—a potentially more dangerous substance than cannabis—among people with pain syndromes [19–22]. SUD treatment programs traditionally focus on complete termination of any drug use, while harm reduction strategies appreciate that recovery is not a linear path for most patients and do not necessarily require complete abstinence from all substances [24]. Indeed, a systematic review that examined reasons for cannabis use among patients living with chronic pain highlighted reducing prescription medication use, namely opioids, as a primary factor motivating patients’ cannabis use [25]. 

Despite the growing acceptance and availability of cannabis products and evidence that cannabis may benefit patients with neuropathic pain, little is known about the perceptions of cannabis use among patients living with chronic pain who also have substance use disorders. The purpose of this qualitative study was to characterize perspectives on cannabis use as a chronic pain therapeutic and a harm reduction strategy among patients diagnosed with chronic pain who were receiving SUD treatment.

## Methods

### Patients and procedures

Patients were recruited from a U.S. Department of Veterans Affairs (VA) medical center’s outpatient SUD treatment program. Patient eligibility was established through telephone interviews and electronic health record (EHR) reviews. Eligible patients were at least 18 years old, participating in the outpatient SUD treatment program, had a chronic musculoskeletal pain diagnosis in their EHR in the preceding five years, and reported an average pain intensity of ≥ 4 on a 0–10 rating scale within the past week and the past 3 months (where 0 = no pain and 10 = worst pain imaginable). Patients who had a cancer pain diagnosis or a pending pain-related disability claim were excluded. All patients provided written informed consent to participate. The study was approved by the Institutional Review Board of the participating VA hospital.

### Treatment setting

The SUD treatment program is located in a U.S. state where laws permit adult use of medicinal and recreational cannabis. However, because the VA is a federal agency, cannabis is not permitted on hospital grounds, and VA clinicians are currently unable to prescribe medicinal cannabis to their patients. The SUD program comprises two tracks. All patients have access to individual and group therapy, delivered by licensed clinical social workers, psychologists, or master’s-trained counselors, as well as addiction medicine services.

The first track focuses on complete cessation of substances for which a use disorder is present and represents the focus of SUD treatment. Patients with polysubstance use may continue to use substances, as long as they commit to a goal of cessation of the substance that is the primary focus of treatment. Patients who continue to use any substances during treatment may speak about their use with program counselors but are asked not to discuss their ongoing use with patients in the program.

This first track, that focuses on substance cessation, permits continued use of cannabis and alcohol, if they are not the primary substances for which a patient is seeking treatment. Some patients may possess medical cannabis “cards,” that serve as a record of the patient receiving a cannabis “prescription” from a non-VA clinician. For patients with a medical cannabis card, SUD treatment program administrators obtain records from the non-VA cannabis-prescribing clinician that document the medical condition(s) for which cannabis is prescribed.

The second track focuses on harm reduction and exploration of patients’ interest in reducing and/or the negative impacts substance use has on patients’ lives. Patients on this second track receive individual motivational interviews from substance counselors and participate in a clinician-led support group composed of only patients in this track. For patients who start on the second track and later determine they seek the complete cessation of a substance, they have the opportunity to switch to the first track. All patients in this study were part of the first track that focused on substance cessation. Nevertheless, this track implements a harm reduction approach, which moves away from the complete cessation of all substances as a primary goal, allowing incremental behavioral changes that may be more sustainable while in SUD treatment [26]. 

### Data collection

Data analyzed in the current study were part of a larger study focused on treating chronic pain among patients with substance use disorders. Forty-six patients were referred to the study based on initial review of the EHR. We were unable to contact seven referred patients, one declined participation, and four missed their scheduled appointments, resulting in 34 patients interviewed. One of the 34 interviewed patients was excluded based on a primary diagnosis of cannabis use disorder, as the purpose of this study was to gather perspectives on cannabis use from patients with non-cannabis primary SUD in this treatment setting (*N* = 33). The study’s principal investigator (PI) completed a semi-structured interview with each patient. Private in-person interviews were audio recorded in an office at the SUD treatment center. Patients received a $30 gift card for completing interviews (see Appendix for the interview guide).

### Data analysis

Interviews were transcribed verbatim by a study team member and reviewed by the PI for accuracy. A codebook was developed by four doctoral-level scientists with training in qualitative methods who independently coded a random sample of five interview transcripts. Codes were developed inductively (i.e., open coded) following conventional content analysis [27]. Each coder “tagged” sections of text with a descriptive label or code utilizing Atlas.ti version 7.

The analysis team randomly assigned primary and secondary coders to each transcript. Transcripts were coded by the primary coder and reviewed by the secondary coder for accuracy. Disagreements between the primary and secondary coders were resolved through team consensus. We then pulled quotes associated with codes that described patients’ use of cannabis generally, and cannabis used for the management of pain specifically. These queries produced reports including data tagged with the identified codes. The analysis team reviewed and sorted the text into categories and identified similarities and differences within and across categories to develop key themes.

## Results

### Cohort characteristics

The sample (*N* = 33) was predominantly male (88%), and White-non-Hispanic (97%) with an average age of 51 years. 33% of patients reported currently or occasionally using cannabis while enrolled in this SUD treatment program to manage pain, pain-related anxiety and sleep issues, while an additional 24% reported no current cannabis use but expressed interest in trying it in the future. The majority (87%) of patients reported having used cannabis at some point in their lives, and 81% of patients reported past or current cannabis use to treat pain specifically. Regarding the primary substances that were the focus of SUD treatment, 70% reported an alcohol use disorder, 18% reported an opioid use disorder, and 12% reported a stimulant use disorder primary diagnosis. The average pain intensity in the past three months reported by patients was 5.6 on the 0–10 pain intensity numeric rating scale (Range: 4–10). All patients had at least one chronic musculoskeletal pain diagnosis (Table 1).

**Table 1:** Primary/secondary SUD, chronic pain diagnoses, current cannabis use/interest

Patient ID *N* = 33	Primary SUD	Secondary SUD	Chronic pain Dx	Current cannabis use/interest
1	Alcohol dependence	Cannabis dependence	Osteoarthritis	No/Interested in CBD use
2	Heroin dependence	Cocaine and Opioid dependence	Rheumatoid arthritis and ankle injury	Yes
3	Alcohol dependence	Stimulant dependence	Osteoarthritis, foot pain and migraines	Yes
4	Alcohol dependence	Cocaine and amphetamine abuse, cannabis and stimulant dependence	Foot, knee, low back pain and sciatica	Yes
5	Opioid dependence	None	Shoulder and lower back pain	No/Interested in CBD use
6	Alcohol dependence	None	Plantar fasciitis	No
7	Alcohol abuse	Cocaine and cannabis abuse, and opioid dependence	Back pain	No
8	Opioid dependence	Cannabis dependence	Tendinitis and myofascial pain	Yes
9	Alcohol dependence	None	Back pain	No
10	Alcohol dependence	None	Low back and shoulder pain	Yes
11	Heroin dependence	Alcohol dependence	Low back pain, spinal stenosis and migraines	No/Interested in future use
12	Alcohol dependence	Opioid dependence	Knee and wrist osteoarthritis	No
13	Methamphetamine dependence	Alcohol dependence	Back pain	No
14	Alcohol dependence	None	Knee osteoarthritis	No
15	Alcohol dependence	Cocaine dependence	Left shoulder pain	No
16	Alcohol dependence	None	Osteoarthritis	No
17	Alcohol dependence	None	Low back pain	No
18	Stimulant dependence	None	Lumbosacral radiculopathy and shoulder pain	No
19	Alcohol dependence	None	Shoulder pain, low back pain and tennis elbow	Yes
20	Alcohol dependence	None	Low back pain and migraines	No
21	Alcohol dependence	None	Knee pain and fibromyalgia	No
22	Alcohol dependence	None	Back, neck and shoulder pain, headaches and dysmenorrhea	Yes
23	Alcohol dependence	Cannabis abuse	Cervical radiculopathy	Yes
24	Alcohol dependence	Amphetamine abuse	Ankle, foot, shoulder and pelvis pain and carpal tunnel	No/Interested in CBD use
25	Alcohol dependence	None	Shoulder pain, cervical radiculopathy and osteoarthritis	No
26	Alcohol dependence	Cannabis abuse	Low back pain	No/Interested in future use
27	Methamphetamine dependence	Opioid abuse	Chronic pain	No
28	Methamphetamine dependence	Alcohol dependence	Low back pain	No/Interested in future use
29	Opioid dependence	None	Ankle, foot and low back pain	Yes
30	Alcohol abuse	None	Spinal stenosis	No
31	Alcohol dependence	None	Neck and shoulder pain	No/Interested in smokeless alternatives
32	Opioid dependence	Alcohol dependence	Low back pain	No
33	Alcohol dependence	None	Shoulder pain	No/Interested in future use

### Primary themes

#### Cannabis for harm reduction, pain, and pain-related anxiety and sleep issues

Some patients perceived cannabis, particularly non-psychotropic CBD, as a harm reducing, non-compromising substance to their treatment program goals and policies. For example, Patient 8 stated, “I’ve been clean off THC for three weeks now, and I’ve been using just straight CBD products…and it’s legal now, it’s not a big deal anymore.” Additionally, Patient 11 stated, “I used [cannabis] during the time that I was sober from heroin and all the other drugs.” For such patients, cannabis products were viewed as acceptable therapeutics while engaged in SUD treatment, as some perceived cannabis as a “softer” alternative compared to “hard” substances like opioids, illicit or prescribed. For example, considering prescribed therapeutics, Patient 23 stated, “Marijuana helps me relax…and I can function, or I take all these pills they want me to take and I’m a zombie,” while Patient 11 stated, “I just know that there are holistic alternatives…marijuana’s one of them,” highlighting the perceived benign profile of cannabis compared to other drugs or medications.

Within this harm reduction setting, patients reported using cannabis for body ache and pain mitigation, as Patient 22 stated, “I do feel that, especially on my neck, [CBD cannabis oil] really helps with the pain.” Patient 13 also noticed that high concentration CBD “really takes the pain away.” Similarly, Patient 2 used cannabis to reduce pain-related anxiety, noting that, after cannabis use, “I’m calmer, I’m not as panicked when [the pain] is really bad.” Patient 3 highlighted the distinction between their use of cannabis for physical pain and pain-related anxiety, mentioning that the “RSO [Rick Simpson Cannabis Oil] is more for the physical pain and if I need something for anxiety…I use the oil pen [smokable cannabis].”

Patients also mentioned using cannabis to induce or sustain sleep in the presence of pain. For example, Patient 18, who struggled to sleep due to pain, stated that after using cannabis, “I was able to go to sleep a lot easier and a lot quicker without all the wrestling around and stuff,” while Patient 11 mentioned that “pain was affecting my ability to fall asleep,” and that cannabis helped them sleep better in general. Further, Patient 1 explained that cannabis helped them stay asleep, “work around the pain,” and not “focus as intently on the pain when under the influence of marijuana,” while Patient 4 mentioned that cannabis “doesn’t make me sleepier, it just helps me stay asleep.”

#### Interest in future cannabis use as a therapeutic agent

Some patients, like patients 1, 5 and 11 were interested in future use, particularly CBD because they believe it to be less psychoactive and a new approach to cannabis use. Moreover, for some who were not currently using cannabis, interest in future cannabis use was framed within the context of addressing their physical pain. Patient 31described a history of smoking cannabis for many years prior to SUD treatment, and having no desire to resume smoking cannabis, while showing interest in using cannabis that did not involve smoking to manage pain, stating, “I want to try the alternative to smoking marijuana [cannabis edibles, oils or topicals for pain]…I’ve never even been to a dispensary… but I’ve been curious to go in.” For this patient and others, the rise in alternative forms of cannabis consumption, the growing popularity of cannabis and the number of cannabis dispensaries in their state was noticeable and piqued their interest.

#### Patient motivations for complete avoidance of cannabis

Some patients reported avoiding cannabis due to unwanted physical and mental effects. For example, Patient 25 described disliking feeling sedated or lethargic, noting that cannabis made “me feel all groggy and sleepy, and I don’t like that.” Patient 30 described avoiding cannabis because it “tends to have me focus on the pain instead of relieving it or making it feel better.” Further, Patient 5 mentioned that cannabis increased their anxiety, stating, “I never liked it [smoking cannabis] personally; it always made me paranoid.” These perspectives highlight contrasting opinions of the effects of cannabis among patients with SUD, some of whom perceive no therapeutic benefit to cannabis.

Additionally, some patients perceived cannabis as a compromising substance that increases the risk of returning to use of substances for which they have a diagnosed disorder. For example, Patient 18 perceived cannabis as a substance that could lead to the abuse of other illicit substances, noting, “I quit everything when I stay sober…[cannabis] could lead me back to doing meth; I try to stay away from all of it.” Additionally, Patient 7 reported that while cannabis “worked fabulously” for their pain, they ceased to use all “substances except what’s prescribed” while in SUD treatment. Due to a fear of returning to use substances targeted for cessation, even when cannabis use is permitted when aligned with a harm reduction strategy, patients may find a strict adherence to suggested SUD treatment plans as paramount.

## Discussion

Nearly 30% of patients diagnosed with chronic pain in this study reported current cannabis use for either pain, pain-related anxiety, sleep issues due to pain, or as a therapeutic alternative while receiving SUD treatment. Understanding that 1 in 10 veterans use cannabis [28], and that more than 1 in 10 veterans have a SUD [29], our findings have broader implications, particularly in states where cannabis use is legal, and in SUD treatment programs that implement a harm reduction approach. Our findings show that all patients who did use cannabis during SUD treatment had either a primary alcohol or opioid dependence diagnosis. Cannabis has shown to be and effective harm reducing therapeutic for both alcohol and opioid SUD treatment [30]. Although cannabis use was permissible if patients progressed toward their non-cannabis SUD treatment goals, several patients who abstained from cannabis use during SUD treatment expressed interest in future therapeutic cannabis use based on recent cannabis science, promotion and laymen’s knowledge.

A growing number of studies examine cannabis’ efficacy for various medical conditions, including pain, with some showing that veterans with chronic pain may choose to use cannabis as a therapeutic [30–34]. While other alternative pain relief therapies for veterans exist, including non-pharmacological approaches like yoga and acupuncture [36, 37], cannabis has shown to be an effective pain reliever that can enhance the quality of life of long term users [38, 39], and serve as a harm reducing substance [40]. 

Some patients who abstained from cannabis while in SUD treatment expressed concerns about cannabis leading them to use the substance they have targeted for cessation, and about the impact of cannabis use on their overall SUD treatment goals. Some patients abstained from cannabis use due to the negative physical or mental side effects they experienced using cannabis, such as lethargy, a heightened sense of one’s pain, anxiety and paranoia. As we see in our findings, the side effects of cannabis can vary among individual patients, and unlike standard pharmacotherapies, cannabis products lack strict regulation resulting in considerable variability in the cannabis strains sold in dispensaries [41]. 

Variability in the potency and the individual mental and physical effects of cannabis complicates patients’ and clinicians’ ability to predict cannabis use healthcare outcomes faithfully. While patients who are prescribed cannabis by a clinician may trust that the products available to them will adequately manage their medical conditions, clinicians lack control over the products patients actually receive from dispensaries. Patients in this study often relied on dispensary staff, friends and heuristic knowledge as motivating factors, and as a risk management strategy when considering the use or non-use of cannabis while in SUD treatment. Unfortunately, no studies have examined SUD treatment outcomes between patients who use and those who do not use cannabis while in SUD treatment. Randomized controlled trials and high-quality observational studies are needed to determine if cannabis use during SUD treatment leads to positive health outcomes or compromises the quality of care SUD patients receive. Nevertheless, our findings expand on existing literature and are a novel step towards longitudinal studies on SUD patient health outcomes regarding cannabis use for harm reduction and pain while in SUD treatment.

### Limitations

This qualitative study has several limitations. First, interviews were conducted with VA patients receiving care in a single SUD treatment program among predominantly male, White non-Hispanic, middle-aged U.S. veterans. As such, findings may not generalize to other settings and populations. Second, most patients were receiving treatment for alcohol use disorder, and findings may not be applicable to populations dealing with largely non-alcohol primary SUD. Third, this is a qualitative study focused on veterans’ perspectives of cannabis use during SUD treatment, and while valuable, our qualitative data preclude quantifying reasons for cannabis use or abstinence. Epidemiologic studies with larger samples are needed to better identify the prevalence of, and reasons for, cannabis use while in SUD treatment.

## Conclusions

This study contributes to the limited knowledge on SUD patient perspectives of cannabis use by exploring those of U.S. military veterans with SUD and chronic pain enrolled in a SUD treatment program. Cannabis use was fairly common among SUD patients who were permitted to use cannabis while in treatment. However, motivations for cannabis use varied as patients used cannabis to alleviate physical pain, mental health issues like anxiety and for problems with sleep due to pain, with many perceiving cannabis as a harm reducing therapeutic. Clinicians, particularly those in the VA Healthcare System and other federally employed providers in states where cannabis is legal, should be prepared to educate patients on the medical evidence, gaps, policies, and federal and state laws regarding cannabis use. However, the ability for such a knowledge exchange remains limited, particularly in federal healthcare settings, due to a lack of comprehensive federal guidance, uncertainty, and fear of policy violations and punishment among clinicians and patients. This further complicates the ability for clinicians to help patients manage risks and make informed healthcare decisions regarding cannabis use.

## Data Availability

The datasets used and/or analyzed during this study are available from the corresponding author upon reasonable request.
